# Engaged Aging: Disparities in Paid and Unpaid Work in Later Life

**DOI:** 10.1093/geroni/igaf122.1073

**Published:** 2025-12-31

**Authors:** Christina Matz, Chin-Yi Su, Julie Miller

## Abstract

As the U.S. population ages, engagement in both paid and unpaid work remains central to older adults’ well-being and societal contributions. However, disparities in access, participation, and the extent to which engagement leads to positive outcomes persist across racial, ethnic, and socioeconomic contexts. This symposium examines how different forms of work—including employment, caregiving, and volunteerism—shape the aging experience, highlighting disparities and identifying opportunities for policy and workplace interventions. The first presentation examines ethnic subgroup differences in volunteerism among Asian American adults aged 50 and older, drawing on nationally representative data to explore how socio-economic and cultural factors shape engagement. The second presentation identifies caregiving typologies among older workers, revealing how caregiving responsibilities interact with employment to impact well-being. The third presentation presents findings from a 2025 nationally representative AARP study on age discrimination among multicultural older workers, documenting high rates of both overt and subtle forms of discrimination, as well as employment transitions driven by financial need. The final presentation examines the effects of productive aging activities—including education, employment, and volunteering—on cognitive health among older Hispanics, considering the moderating role of genetic inheritance. By examining these topics through diverse lenses, this symposium advances understanding of how disparities in work, caregiving, volunteering and education, shape later-life outcomes. Julie Miller, AARP Thought Leader, will synthesize findings and and discuss implications for workplace policies, financial planning, and social programs aimed at fostering greater equity in aging experiences.

